# Signaling pathways in systemic lupus erythematosus and therapeutic implications

**DOI:** 10.3389/fimmu.2025.1735301

**Published:** 2026-01-07

**Authors:** Lili Balogh, Viktor Gyula Kovács, György Nagy, Tamás Németh

**Affiliations:** 1 Department of Physiology, Semmelweis University School of Medicine, Budapest, Hungary; 2 MTA-SE “Lendület” Translational Rheumatology Research Group, Hungarian Academy of Sciences and Semmelweis University, Budapest, Hungary; 3 Department of Rheumatology and Immunology, Semmelweis University, Budapest, Hungary; 4 Heart and Vascular Center, Semmelweis University, Budapest, Hungary; 5 Department of Genetics, Cell- and Immunobiology, Semmelweis University, Budapest, Hungary; 6 Department of Internal Medicine and Oncology, Semmelweis University, Budapest, Hungary

**Keywords:** receptors, signaling, small molecule inhibitors, systemic lupus erythematosus, targeted therapy

## Abstract

Systemic lupus erythematosus (SLE) is a classic immune complex-mediated autoimmune disease that arises from the loss of tolerance to specific self-antigens (such as nuclear antigens). It can cause severe organ damage, leading to lifelong disabilities and potentially life-threatening manifestations. While some new therapeutic options for SLE have been approved in recent years, many patients remain refractory to treatment, making it difficult to achieve remission or low disease activity. While the focus of novel therapies in the pipeline mainly lies on (cell-depleting) biological or cell-based therapies, the better understanding of the disease pathogenesis has revealed several intracellular targets, the inhibition of which could nicely contribute to a more effective treatment strategy in SLE. We provide a concise summary of key receptor signaling pathways – including immuno-, Toll-like and type I interferon receptors – involved in the pathogenesis of SLE. We put special emphasis on intracellular molecules with their current or potential role as therapeutic targets in the control of this devastating disorder. Overall, our aim was to draw attention to the field of signal transduction therapy in SLE, which already has a partial role in the current treatment guidelines, but could have more beneficial contributions to the future therapy of this autoimmune disorder.

## Introduction

The hallmark of systemic lupus erythematosus (SLE) is the autoimmune failure that results in a loss of immune tolerance toward self-antigens, including dsDNA, histones, and the Smith antigen (1). The incidence of the disease is approximately 5 per 100 000 person-years (meaning 0.4 million new cases annually), while the prevalence is estimated to be 43 per 100 000 people (resulting in a 3.4-million population), however, the distribution varies with geographical locations (2). Due to hormonal factors, 85% of the patients are women, many at the childbearing age (2). There are several non-targeted and targeted therapies, which are used in the management of the disease, from the small molecule hidroxi-chloroquine to biological therapies like belimumab, anifrolumab or rituximab (3). Meanwhile, there are several orally available small molecules targeting signal transduction, which are being tested in clinical studies.

B cells are crucial players in the pathogenesis, due to their capability to present autoantigens through MHC class II molecules, to produce cytokines or to exert other effector functions, while being the origin of autoantibody-producing plasma cells (4). B cell-targeted therapies like the anti-BAFF antibody belimumab and the CD20-specific rituximab are widely used in the treatment of SLE, while novel B cell depleting agents like obinutuzumab are also arriving to the everyday clinical practice (5). Meanwhile, B cell- or plasma cell-focused cellular therapies, namely anti-CD19 or anti-BCMA chimeric antigen receptor- (CAR-) T cell therapies showed promising and long-lasting effects in selected cases of severe SLE with the possibility of sustained drug-free remission (6–9). However, the current cost and the required infrastructure of ex vivo CAR-T cell therapy potentially excludes its wider use in SLE patients, while novel approaches may become available to more patients (10). Meanwhile, B cell receptor signal transduction is overactivated and the negative regulators are defective in many SLE patients, highlighting several possible intracellular targets for future therapies (11).

In addition to B cells, effector T cells are known to drive the inflammatory reaction, while the number and function of regulatory T cells are simultaneously reduced (12). Effector T cells have a lower activation threshold of their T cell receptors, which results in overactivation leading to B cell interaction and accelerated migration by the help of integrins to tissues (13). However, T cell exhaustion may have an important contribution to disease remission (14).

Several types of the pattern recognition receptors Toll-like receptors (e.g. TLR3–5 or TLR7-9) can be found in immune cells (e.g. in macrophages, in dendritic cells or in B cells) and have been shown to be involved in the pathogenesis (15). Besides extracellular antigens, Toll-like receptors can also detect self molecules like oligonucleotides, leading to the production of type I interferons, which are considered to be central cytokines in the pathogenesis (15). Toll-like receptor signaling molecules have been considered to be potential targets in the control of SLE (15).

In addition to B and T cells, Toll-like receptor expressing plasmacytoid dendritic cells stand in the center of the inflammatory process and produce high amounts of type I interferons (16). Highlighting the essential role of type I interferons in the pathogenesis, anifrolumab, a monoclonal antibody against the type I interferon receptor subunit 1 (IFNAR1) has been approved by the US Food and Drug Administration (FDA) and the European Medicines Agency (EMA) for non-renal manifestations (and is being investigated in lupus nephritis). Autoantigen and autoantibody containing “SLE-specific” immune complexes are linked to type I interferon production of plasmacytoid dendritic cells, but can also activate classical myeloid cells (like macrophages, dendritic cells or neutrophils) through their Fc receptors (17). The importance of Fcγ receptors in SLE is highlighted by the fact that some genetic variations of their genes have been linked to the development of SLE (18).

**Figure 1:**
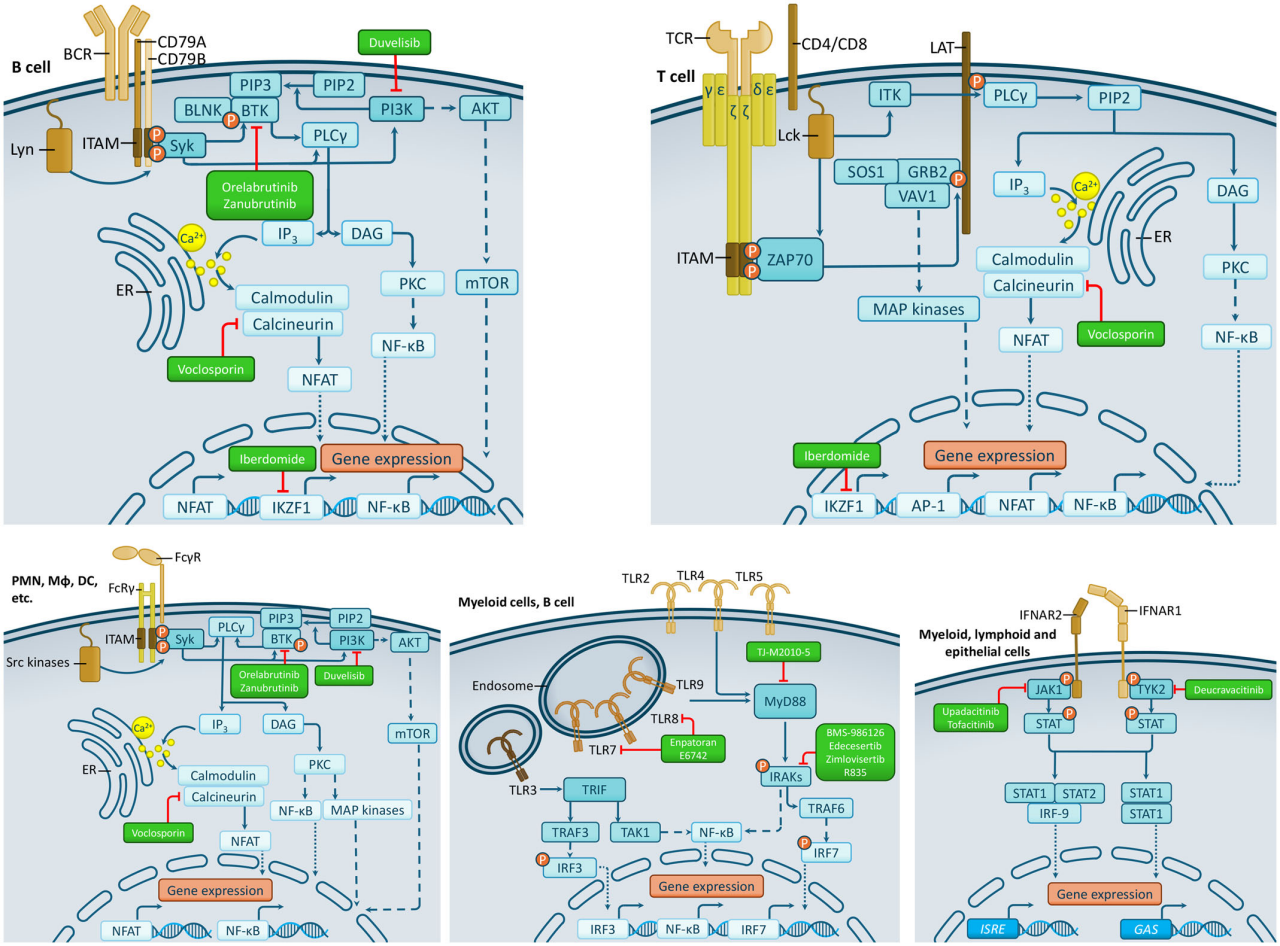
The most important signaling pathways with significant therapeutic potential. The schematic depiction of signal transduction pathways of B cell, T cell, Fc, Toll-like and Type I interferon receptors are shown with specific cell expression patterns. Abbreviations: AP-1, Activator protein-1; BCR, B cell receptor; BLNK, B cell linker; BTK, Bruton’s tyrosine kinase; DAG, diacylglycerol; DC, dendritic cell; ER, Endoplasmic reticulum; ERK, Extracellular signal-regulated kinase; FcγR, Fcγ receptor; FcRγ, Fc receptor γ-chain; GAS, Growth Arrest Specific; GRB2, Growth factor receptor-bound protein 2; IFNAR, interferon-α/β receptor; IKK, IκB kinase; IKZF1, Ikaros family zinc finger protein 1; IP3, inositol 1,4,5-trisphosphate; IRAKs, Interleukin-1 receptor-associated kinases; IRF, Interferon regulatory factor; ISRE, Interferon-Stimulated Response Element; ITAM, Immunoreceptor tyrosine-based activation motif; ITK, interleukin-2-inducible T cell kinase; JAK, Janus kinase; JNK, c-Jun N-terminal kinase; LAT, Linker for activation of T cells; MAPK, mitogen-activated protein kinase; mTOR, Mammalian target of rapamycin; MyD88, Myeloid differentiation primary response 88; Mϕ, macrophage; NFAT, Nuclear factor of activated T cells; NF-κB, Nuclear factor κB; PI3K, phosphatidylinositol 3-kinase; PIP2, Phosphatidylinositol 4,5-bisphosphate; PIP3, Phosphatidylinositol 3,4,5-trisphosphate; PLCγ, Phospholipase Cγ; PKC, Protein kinase C; PMN, polymorphonuclear leukocytes (neutrophils); SOS, Son of Sevenless; STAT, Signal Transducer and Activator of Transcription; TAK1, Transforming growth factor-β-activated kinase 1; TCR, T cell receptor; TLR, Toll-like receptor; TRAF, Tumor necrosis factor receptor-associated factor; TRIF, TIR Domain-containing adaptor inducing IFN-β; TYK2, Tyrosine kinase 2; ZAP70, Zeta-chain associated protein of 70 kDa.

In this review, we give a short overview of the most important receptor signaling pathways driving inflammation during the pathogenesis of SLE with a special focus on the therapeutic implications of signal transduction molecules (**Figure 1**, **Table 1**). These novel therapeutic agents may serve as new options between the hidroxi-chloroquine and the biological therapy arms in the future treatment algorithms of non-renal lupus in contrast to the current one (3).

**Table 1:** Signal transduction therapy in SLE: current medications and potential drug candidates.

Receptor	Target	Drug	Status (approved or clinical study phase)	References
B cell receptor (BCR)	BTK	orelabrutinib	phase 1/2	NCT04305197 (21),
		zanubrutinib	phase 2	NCT04643470
		BI-BTK-1	preclinical	(23–25)
	PI3K	duvelisib	preclinical	(26)
	Ikaros (IKZF1), Aiolos (IKZF3)	iberdomide	phase 2	(29)
T cell receptor (TCR)	Lck	atorvastatin	preclinical	(32)
	calcineurin	voclosporin	approved	(33)
	GRK2	CP-25	preclinical	(35)
	Ikaros (IKZF1), Aiolos (IKZF3)	iberdomide	phase 2	(29)
Toll-like receptor (TLR)	TLR7/8	enpatoran	phase 2	NCT05162586 (40),
	TLR7/8	E6742	phase 1/2	NCT05278663 (41),
	MyD88	TJ-M2010-5	preclinical	(42)
	IRAK4	BMS-986126	preclinical	(43)
		edecesertib	phase 2 (cutaneous lupus erythematosus)	NCT05629208
		zimlovisertib	phase 1	(39)
	IRAK1 and IRAK4	R835	phase 1	(39)
Type I interferon receptor (IFNAR)	JAK1	upadacitinib	phase 2	NCT03978520 (45),
	TYK2	deucravacitinib	phase 2	NCT03252587 (46),
	JAK1 and JAK3	tofacitinib	retrospective cohort study	(49)

BCR, B cell receptor; BTK, Bruton’s tyrosine kinase; GRK2, G protein-coupled receptor kinase; IFNAR, Interferon α/β receptor; IKZF, Ikaros family zinc finger protein; IRAK4, Interleukin-1 receptor-associated kinase 4; JAK, Janus kinase; PI3K, phosphatidylinositol 3-kinase; TCR, T cell receptor; TLR, Toll-like receptor; TYK2, Tyrosine kinase 2.

## Signal transduction and potential small molecule based-therapy of systemic lupus erythematosus

### B cell receptor signaling

B cell activation occurs largely through B cell receptors (BCRs), B cell activating factor receptors (BAFF-Rs) and Toll-like receptors (TLRs). BCR can recognize foreign antigens, and upon ligand-binding a cross-linking and oligomerization of the receptor occur (19). The ligand binding receptor unit makes a complex with the CD79A/CD79B chains, which carry immunoreceptor tyrosine-based activation motifs (ITAMs) that get phosphorylated by Src kinases, like Lyn, and recruit downstream kinases like the Syk and the Bruton’s tyrosine kinase (BTK) (20). During the diverse phosphorylation cascade, the activation of phospholipase Cγ2 (PLCγ2) results in Ca^2+^-release – which enables the functions of Ca^2+^-dependent enzymes like calcineurin – and the translocation of nuclear factor kappa B (NF-κB), meanwhile the phosphorylation of phosphoinositide 3-kinase (PI3K) activates the protein kinase B (AKT) pathway thereby contributing to cell survival (**Figure 1**) (20). Altogether, signaling through BCR is essential for survival, proliferation and many effector cell responses (19).

BTK is becoming an appealing therapeutic target in several autoimmune diseases, and it has an important role in the downstream signaling of the BCR-mediated pathway. *Orelabrutinib* seemed to be effective in SLE patients: higher proportion of patients achieved the SLE responder index (SRI)-4 response at week 12 in the orelabrutinib groups compared to placebo (21). The effect was even more robust in patients with a higher disease activity in this phase 1/2 clinical study (NCT04305197) (21). Orelabrutinib also had a favorable effect on the remission rate of arthritis, effectively reduced anti-dsDNA titers and increased complement levels (21). In addition, it was a well-tolerated therapy: lymphocyte count reduction, anemia, petechia and upper respiratory tract infections occurred slightly more frequently in orelabrutinib-treated patients than in the placebo group (21). What makes orelabrutinib a promising choice over previously tested BTK-inhibitors (that were found to be non effective in SLE studies) is its very high potency, near 100% target occupancy and maybe its selectivity (22). It is an irreversible BTK inhibitor (as a result of its covalent binding) and its inhibitory effect persists for 24 hours after administration (22). Newer BTK inhibitors like *zanubrutinib* is currently undergoing a phase 2 clinical trial in patients with lupus nephritis (NCT04643470), while *BI-BTK-1* showed promising preclinical results in a murine model of lupus nephritis (23–25).

*Duvelisib* is a PI3K-inhibitor, which decreased the autoantibody-levels in a murine lupus model, while having a beneficial effect on IgG deposition in the kidney and the degree of glomerulonephritis in mice (26).

Ikaros (IKZF1) and Aiolos (IKZF3) belong to the Kruppel transcription factor family. Ikaros has an important role in the development of lymphoid cells (27). Both Ikaros and Aiolos showed to be risk factors in SLE (28). *Iberdomide* is an orally available cereblon modulator that promotes the degradation of the transcription factors Ikaros and Aiolos. At a dose of 0.45 mg, iberdomide-treated SLE patients had a higher SRI-4 response rate than the placebo group (29). Adverse events – most commonly urinary and upper respiratory tract infections or neutropenia – occurred more frequently in the iberdomide-treated patients (29). However, longer clinical trials are needed to provide a more accurate answer to the question of safety (29).

### T cell receptor signaling

The T cell receptor (TCR) is capable of recognizing antigens associated with the major histocompatibility complex (MHC) (30). TCR is a heterodimeric receptor consisting of TCRα and TCRβ chains in αβ T cells, and it is assembled with the CD3 complex proteins (CD3γ, δ, ϵ, and ζ chains), where ITAM regions can be found. The ITAM gets phosphorylated by the Src kinase Lck and ZAP-70 (the T cell-equivalent of Syk) is recruited (30). ZAP-70 phosphorylates the linker for activation of T cells (LAT) and other molecules, which mediate downstream signaling: leading to the activation of transcription factors like the nuclear factor of activated T cells (NFAT) through calcineurin, NF-κB and AP-1 (**Figure 1**) (31). *Atorvastatin* modifies the compositions of lipid rafts resulting in the disruption of colocalization of Lck and CD45 in the lipid raft, leading to the reduction of active Lck. In SLE patients, atorvastatin reduced the IL-10, IL-6 and T cell levels (32).

The novel oral calcineurin-inhibitor *voclosporin* prevents the formation of the calcineurin-calmodulin complex, thereby inhibiting the effect of the calcineurin signaling pathway in T cells (33). Following successful clinical trials, voclosporin was approved for the treatment of lupus nephritis. T cells are major sources of important cytokines during autoimmune inflammation, where the G protein–coupled receptor kinase-2 (GRK2)-dependent development of the TCR-CXCR4 complex is important (34). The pharmacological inhibition of GRK2 using *CP-25* ameliorated the development of pristane-induced lupus, resulting in decreased antibody-production and an improved histopathological phenotype (35).

The above mentioned Aiolos and Ikaros inhibitor iberdomide potentially also acts on T cell receptor signaling in SLE patients (29).

### Fc receptor signaling

The signal transduction of Fc receptors shows many similarities to the signaling of the other two immunoreceptors, namely the B cell and the T cell receptor. When the autoantigen-autoantibody immune complex binds to an activating Fcγ receptor, the ITAM region gets phosphorylated by Src-family kinases (36). These proteins recruit and phosphorylate Syk, which directly or indirectly activates several downstream molecules, including the MAP kinases and NF-κB leading to transcriptional changes, the PI3K or the Bruton’s tyrosine kinase (**Figure 1**) (36). As we mentioned above, Fc receptor signaling overlaps with the signal transduction of the other two immunoreceptors at several aspects, therefore some of the therapies mentioned above (e.g. BTK inhibitors) may also contribute to the improvement of SLE symptoms through the inhibition of Fc receptor signaling.

### Toll-like receptor signaling

Toll-like receptors (TLRs) – as pattern recognition receptors (PRRs) – get activated by specific pathogen-associated molecular patterns (PAMPs) and lead to first-line host defense cell responses (15). Some of these receptors induce cell activation against extracellular microbial agents, while others are located intracellularly and sense RNA or DNA fragments (15). As circulating nucleic acid-containing complexes can stimulate plasmacytoid dendritic cells (pDCs) in systemic lupus erythematosus, some intracellular Toll-like receptors (like TLR3, TLR7, TLR8, TLR9) and their signaling pathways could be potential therapeutic targets (37). Downstream from the receptor-ligand interaction, several intracellular proteins get activated, like MyD88 or IL-1R-associated kinases (IRAKs) (**Figure 1**) (15). Interestingly, patients with MyD88- and IRAK-deficiency are protected against autoreactive autoantibody production (38). One of the major consequences of TLR signaling is the increased type 1 interferon (IFN)-production through transcriptional upregulation of IFN-related genes (38). The inhibition of signal transduction of Toll-like receptors can be approached in two main ways: 1) preventing the binding of TLR to its ligand (by hydroxi-chloroquine, oligonucleotide-based antagonists, small molecules or monoclonal antibodies) or blocking the downstream cascade (by small molecules inhibiting IRAKs, molecules stimulating IRAK degradation or molecules targeting adaptor proteins) (39). The small molecule TLR7/8 inhibitor, *enpatoran* reached significant improvements in the disease activity of cutaneous lupus erythematosus (CLE) patients compared to placebo in a phase 2 clinical trial with an acceptable safety profile (NCT05162586) (40). *E6742*, a dual TLR7/8 antagonist resulted in a suppression of the interferon gene signature responses, while showing promising preliminary efficacy signals in SLE patients in a phase 1/2 study (NCT05278663) (41). Meanwhile, E6742 seemed to be well-tolerated, since the incidence of adverse events was 58.5% in the E6742-treated groups in contrast to 66.7% in connection with the placebo (41). In contrast to the central role of MyD88 in the signaling process, only promising *in vitro* results are available for the MyD88 inhibitor, *TJ-M2010-5*, which was able to reduce the activation of lupus-like B cells by preventing proliferation and antibody production (42). In several experimental lupus models, the inhibition of IRAK4 with a highly selective inhibitor (by *BMS-986126*) resulted in a reduction in skin and kidney manifestations in a dose-dependent manner (43). Several inhibitors of the IRAK enzymes – like *zimlovisertib, edecesertib* or *R835* – have now completed successful phase 1 trials, where mainly their acceptable safety profiles were determined, but a few efficiency-related effects were also included, such as inhibition of cytokine release in the case of R835 treatment (39). There is already an ongoing phase 2 trial with edecesertib in cutaneous lupus erythematosus patients (NCT05629208).

### Type 1 interferon receptor signaling

Type 1 interferons (IFNs) specifically bind to their heterodimeric receptor, which contains a high affinity (IFNAR2) and a low affinity (IFNAR1) receptor chain (44). The binding of type 1 IFNs to their receptors induces the intracellular signaling through Janus-kinases (JAKs). IFNAR2 is associated with JAK1, while IFNAR1 leads to the activation of another JAK, namely Tyrosine kinase 2 (TYK2) (44). Activated JAKs go through autophosphorylation, then phosphorylate the IFN receptor and recruit Signal transducer and activator of transcription proteins (STATs). This leads to the nuclear translocation of STATs, which triggers the activation of interferon-stimulated genes (**Figure 1**) (44). The JAK1 inhibitor *upadacitinib* showed significant improvements in the disease activity in SLE patients: 54.8% of upadacitinib-treated patients achieved SRI-4 response rate and were able to reduce the daily glucocorticoid dose to a maximum of 10 mg by week 24 compared to the 37.5% in the placebo group. Upadacitinib also reduced the frequency of flares, while novel safety concerns were not detected in the upadacitinib group in this phase 2 clinical trial (NCT03978520) (45). Upadacitinib is currently undergoing a phase 3 trial, where it is administered orally once daily (NCT05843643). In another phase 2 clinical study, the TYK2-inhibitor *deucravacitinib* brought promising results: significantly higher proportion of the treated groups achieved the primary endpoint compared to the placebo (NCT03252587); while the incidence of serious adverse events was comparable between the treated and placebo groups (46). Two currently ongoing phase 3 trials will help to more accurately determine the effect of deucravacitinib in SLE patients (NCT05617677, NCT05620407) (47). Moreover, in the MRL/lpr lupus model, the JAK1/JAK3 inhibitor *tofacitinib* ameliorated renal manifestations, while decreasing plasma anti-dsDNA levels and proteinuria (48). Tofacitinib also demonstrated significant therapeutic potential in SLE patients with arthritis in a retrospective cohort study, where tofacitinib resulted in a reduction in serum Il-6 levels and T cell activation (49). While there was a higher incidence of infections in connection with tofacitinib therapy, no severe adverse event was detected (49).

## Discussion and concluding remarks

While some new therapies have been approved by the FDA and/or by the EMA during the past few years, there are still a significant number of SLE patients (approximately 30% in the case of lupus nephritis), who do not respond well enough to the available medications (50). This means that novel therapeutic options are needed.

B cell- or plasma cell-depletion by mono- and bispecific antibodies or by CAR-T cells is one of the main focus, while other B cell-targeting biological therapies are still in the pipeline (5–9, 51, 52). The bispecific antibody teclistamab is a very potent plasma cell-depleting agent in refractory SLE cases with a good accessibility, however, it has a significant infection risk and can cause cytokine release syndrome. The current ex vivo form of CAR-T cell therapy is very expensive, requires a complex infrastructural background (a Good Manufacturing Practice /GMP/ facility with strict quality control), needs conditioning lymphodepletion with chemotherapy (like fludarabine and cyclophosphamide), which can cause toxicity, can affect hematopoiesis and fertility, while CAR-T cells can provoke cytokine release syndrome (or neurotoxicity), leaving this option exclusively for severe, therapy-resistant patients (6, 7). However, CAR-T cell therapy can cause a reset in the dysregulated immune system and may offer the possibility of sustained drug-free remission in autoimmunity (53). Nevertheless, B and plasma cell-depletion in general has some limitations (54).

Meanwhile, it is well known that abnormal immuno-, Toll-like and type I interferon receptor signaling can be present in SLE patients with several pathway components that could serve as drug targets (**Table 1**). While some current (targeted) small molecule medications (like voclosporin) are already important additional pillars of the treatment of renal manifestations, there are many potential other small molecule drug candidates (which target important participants of the signal transduction routes of the immuno-, Toll-like or type I interferon receptors) that could represent further therapeutic options (55). The advantages of targeted small molecules (i.e. signaling therapies) in contrast to monoclonal antibodies rely on their oral administration, easier production and handling, lower immunogenicity and a combination potential (56). However, the pharmacokinetics can be affected by several factors (e.g. gastrointestinal motility), the infection risk can be significant and these medications need higher compliance from the patient’s side.

Novel signal transduction therapies could help to decrease the number of therapy-refractory SLE cases, could serve as novel therapeutic options between the hydroxi-chloroquine and the biological therapy arms in the future treatment algorithms of non-renal lupus, but could also work in concert with the current available treatment strategies (3).
